# Superficial Intradermal Injections of Cohesive Polydensified Matrix Hyaluronic Acid Fillers for the Improvement of Facial Pores and Skin Quality: A Split‐Face Randomized Study

**DOI:** 10.1111/jocd.70209

**Published:** 2025-04-30

**Authors:** Thanachat Rutnumnoi, Pasita Palakornkitti, Tanaporn Anuntrangsee, Tanat Yongpisarn, Nawara Sakpuwadol, Vasanop Vachiramon

**Affiliations:** ^1^ Division of Dermatology, Faculty of Medicine Ramathibodi Hospital Mahidol University Bangkok Thailand; ^2^ Division of Dermatology, Department of Internal Medicine, Faculty of Medicine Naresuan University Phitsanulok Thailand

**Keywords:** dermal filler, facial pores, injectables, rejuvenation, skin booster

## Abstract

**Background:**

Various therapeutic options have been introduced for enlarged facial pores including low cross‐linked hyaluronic acid (HA) fillers. Newer formulations of HA‐based dermal fillers are continuously introduced into the market, but their effectiveness in reducing enlarged facial pores has not yet been determined.

**Objectives:**

To compare the efficacy of HA‐based dermal fillers (Cohesive Polydensified Matrix HA filler; CPM‐HA20) versus CPM‐HA20 with glycerol (CPM‐HA20G) in terms of minimizing enlarged facial pores and skin quality improvement.

**Methods:**

Thirty subjects with enlarged facial pores were enrolled in this randomized, double‐blinded, split‐face study. Participants were randomly assigned to be injected with 1 mL of CPM‐HA20 filler on one side of their medial cheek and 1 mL of CPM‐HA20G on the contralateral side for 3 sessions spaced 4 weeks apart. Pore volume was objectively measured by an Antera 3D. Skin biophysical properties were evaluated. Participant satisfaction and adverse events were recorded.

**Results:**

Twenty‐nine participants completed the study. Both treatment groups showed a reduction in the mean pore volume from the baseline through Week 32. The CPM‐HA20G treated side showed a 24.2% higher reduction in mean pore volume from baseline compared to the CPM‐HA20 treated side (*p* = 0.038). Both treatment groups showed improvement in skin hydration from baseline to Week 32. There was no significant difference in patient satisfaction between the CPM‐HA20G and CPM‐HA20 treated sides. Only mild adverse events such as pain, edema, and bruising were reported.

**Conclusion:**

Three‐monthly injections of CPM‐HA20G and CPM‐HA20 were effective in minimizing enlarged face pores and improving skin hydration. CPM‐HA20G demonstrated superior efficacy in terms of pore size reduction. Adverse events were generally mild and tolerable.

## 1 Introduction

In recent years, there has been a growing interest among individuals in how to get good skin quality. This can be attributed to the general perception that good skin quality indicates a strong, youthful, and attractive appearance. There are currently no established criteria for defining skin quality. However, numerous conceptual frameworks for assessing skin quality have been presented [1, 2]. A consensus among all research is that pores, which are the landmark structure of pilosebaceous units, play a crucial role in determining skin quality. Pore size can be attributed to factors such as age, gender, and ethnicity. Age is associated with an increase in enlarged facial pores, particularly in the 30s and 40s. Face‐specific variations exist in the quantity of enlarged pores (e.g., nose, forehead, cheek) [3]. Asians exhibit smaller pore size and less pronounced epidermal architectures surrounding facial pores in comparison to Hispanics and African Americans [4, 5]. However, excessive sebum production, decreased skin elasticity, and larger hair follicles are the three main causes that contribute to enlarged facial pores. In addition, sex hormones, skincare routines, and persistent and recurrent acne can all have an impact on pore size [6, 7, 8].

Presently, the available treatments for enlarged facial pores include topical retinoids, chemical peeling, oral antiandrogen, energy‐based devices, and injectables [6, 9, 10]. Dermal fillers, particularly those based on hyaluronic acid (HA), are frequently used to rejuvenate the skin and reduce enlarged facial pores [11, 12]. Currently, substantial progress has been made in the formulation of innovative soft‐tissue HA fillers that are specifically formulated to enhance the quality of the skin. The revitalizing properties of a glycerol‐containing low cross‐linked HA filler have been validated in three separate studies [13, 14, 15]. These studies have demonstrated improvements in skin hydration, elasticity, firmness, roughness, fatigue, skin tone, and radiance. Due to the improvement of skin elasticity, we hypothesize that it should work on enlarged pores. However, no study has been performed to evaluate its efficacy in terms of minimizing enlarged facial pores. Therefore, this study was conducted to compare the efficacy of low cross‐linked dermal HA filler containing glycerol with conventional dermal HA filler in reducing enlarged facial pores.

## 2 Materials and Methods

### 2.1 Study Design

This is a prospective, randomized, double‐blinded, split‐face study conducted at a university‐based hospital (Ramathibodi Hospital, Mahidol University, Bangkok, Thailand) from October 2022 to June 2023. This study protocol conformed to the guidelines of the Declaration of Helsinki and was approved by the Institutional Review Board of Human Rights Related to Research involving Human Subjects, Faculty of Medicine Ramathibodi Hospital, Mahidol University, Thailand (Protocol number MURA2022/151). Written informed consent was obtained from all participants before study initiation.

### 2.2 Study Participants

A total of 30 participants aged 18 years or older had received the research information and voluntarily signed the informed consent form. They were recruited from public announcements through social media. The exclusion criteria were participants with the following: (i) infection, inflammatory lesion, or wound around the injection site; (ii) history of skin cancer, keloids, or hypertrophic scars; (iii) uncontrolled active acne vulgaris; (iv) previous cutaneous surgery at the injection site within 6 months and previous mid‐face HA injection within 1 year prior to the study; (v) recurrent cutaneous herpes infection; (vi) pregnancy and lactation.

### 2.3 Treatment Protocol

**FIGURE 1:**
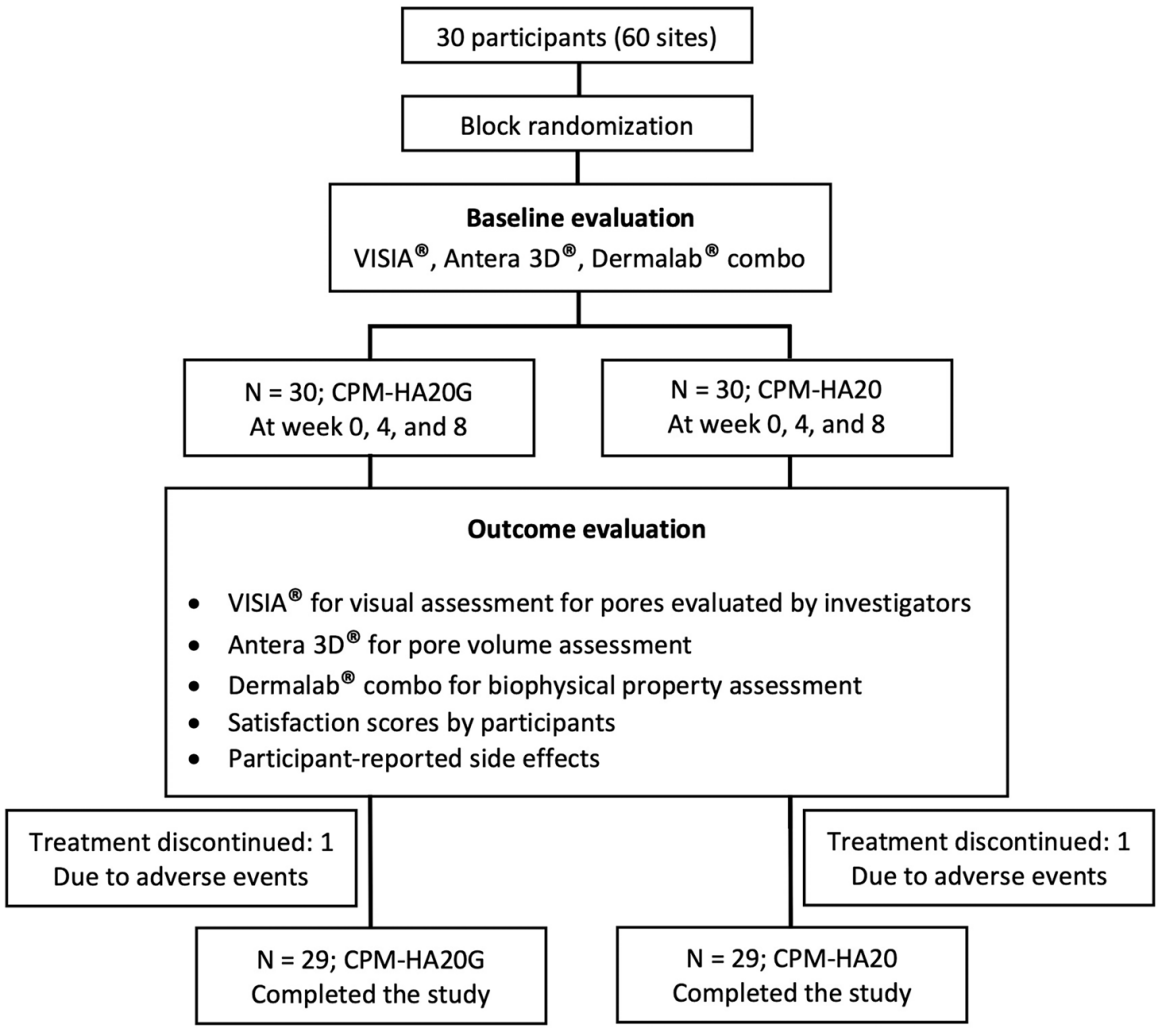
Protocol flow chart.

Before the treatment, participants were instructed to clean their facial skin with a neutral cleanser or water. All participants were randomly assigned to be treated with 1 mL of intradermal injection of cohesive polydensified matrix HA filler containing glycerol (Cross‐linked sodium hyaluronate gel 20.0 mg/mL and glycerol 17.5 mg/mL; CPM‐HA20G; Belotero Revive, Merz Pharmaceuticals GmbH, Frankfurt, Germany) with 0.05 mL per injection point for a total of 20 injection points using 30 G ½ needles (0.3 × 13 mm) in the medial cheek on one side and 1 mL of CPM‐HA20 (Belotero Soft, Merz Pharmaceuticals GmbH, Frankfurt, Germany) with the same protocol on the contralateral side over three consecutive sessions at Day 0, Week 4, and Week 8. The protocol flow chart is shown in Figure 1.

### 2.4 Outcome Evaluation

Baseline demographic data were recorded, and standard facial photographs were taken by VISIA (Canfield Scientific, Parsippany, NJ) for evaluation at baseline Week 0, Weeks 4, 8, 12, 16, 20, and 32. All photographs were taken from the front and 45° from the left and right sides in the same positions.

For objective assessment, pore volume (mm^3^) was evaluated by Antera 3D (Miravex Limited, Dublin, Ireland) with the medium filter to detect the size of the pores in up to 1 mm^2^. Biophysical parameters were assessed by DermaLab combo (Cortex Technology, Hadsund, Denmark) at baseline (Week 0), Weeks 4, 8, 12, 16, 20, and 32. These parameters included skin hydration, transepidermal water loss (TEWL), and elasticity properties. The average value of three measurements was reported in each parameter. For skin hydration, the PIN probe with eight contact pins was used to measure the conducting properties of the skin. The average skin hydration output was reported in the unit of micro‐Siemens, μS). The TEWL probe was used to determine the relative skin surface humidity by converting the density gradient of water evaporation from the epidermis into a TEWL value in g/m^2^/h. Elasticity measurements were performed with a suction probe with a double adhesive sticker. Based on the elevation phase and retraction phase, two parameters were calculated as viscoelasticity (mega‐Pascal, MPa) and retraction time (milli‐seconds, ms).

Subjective assessment was evaluated byone‐blinded dermatologist in terms of improvement on a five‐point scale of pore grading score by criteria of visual assessment for pores ranging from 1 to 4 (1 stand for small and invisible pores and 4 for large and obvious pores) [16]. The Global Aesthetic Improvement Scale (GAIS) was assessed by participants using seven‐point Likert scale from grade +3 “very much improved” to grade −3 “very much worse.”

### 2.5 Adverse Events

In all follow‐up visits, the participants were asked to report any adverse events encountered. Adverse events were recorded as binary outcomes (yes or no), including pain, bruising, hematoma, palpable dermal nodule, itching, and swelling. Pain score was recorded by participants immediately post‐injection at each treatment session using a visual analog scale (VAS, 0–10).

### 2.6 Statistical Analysis

Statistical analysis was performed using STATA/SE version 14.2 (STATA CorpLLC, College Station, TX). Baseline characteristics of participants, that is, age, were described using mean and standard deviation (SD). The categorical characteristics, that is, sex, Fitzpatrick skin type (FPT), were described as frequency and/or percentage. For continuous outcomes, that is, hydration and pore volume, they were summarized by visit using mean and standard deviation, while pore grading score, which is an ordinal outcome, was summarized by visit using median and interquartile range (IQR).

For comparison of the outcomes change (increment/reduction) at each visit from baseline and overall change between groups, the multilevel mixed‐effect linear regression was used to analyze the continuous outcomes (i.e., hydration increment and pore volume reduction). Multilevel mixed‐effect ordered logistic regression was used to analyze the ordinal outcomes that is, pore grading score reduction by physician, and GAIS by participants. The multilevel mixed‐effect models consider the correlation of repeated measurements of the outcomes over time within individual participants into account the models' estimation. A *p*‐value of 0.05 or less was considered statistically significant.

## 3 Results

Thirty participants were enrolled as shown in Figure 1. Participants were randomized to be treated with CPM‐HA20G and CPM‐HA20 on each side of the face for three consecutive sessions at Day 0, Week 4, and Week 8. One participant was withdrawn from the study after Week 4 due to inconvenient follow‐up and was excluded from the efficacy analysis.

### 3.1 Participant Demographics

A total of 29 participants, 25 female (86%) and 4 male (14%) participants, completed the study. The mean age of participants was 36 ± 8 years. One participant (3%) had FPT II, 24 participants (83%) had FPT III, and four participants (13%) had FPT IV. Eight participants (28%) had a history of previous mid‐face HA filler injection. The demographic data are shown in Table 1.

**TABLE 1:** The participant demographic data.

Characteristics	Data
Number of participants	29
Mean age	36 ± 8 years
Gender	
Male	4 (13.8%)
Female	25 (86.2%)
Fitzpatrick skin type	
II	1 (3.4%)
III	24 (82.8%)
IV	4 (13.8%)
Smoking	2 (6.9%)
Previous history of filler at mid‐face (more than 12 months ago)	8 (27.6%)

Abbreviation: SD, Standard deviation.

### 3.2 Pore Volume

**FIGURE 2:**
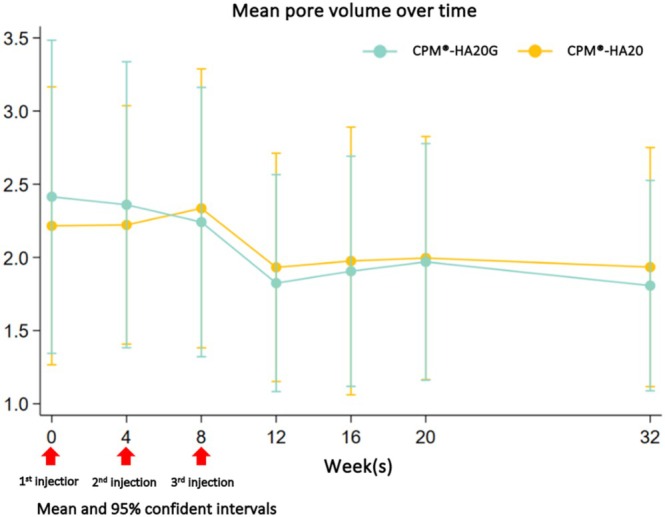
Mean pore volume over time by Antera 3D®.

**FIGURE 3:**
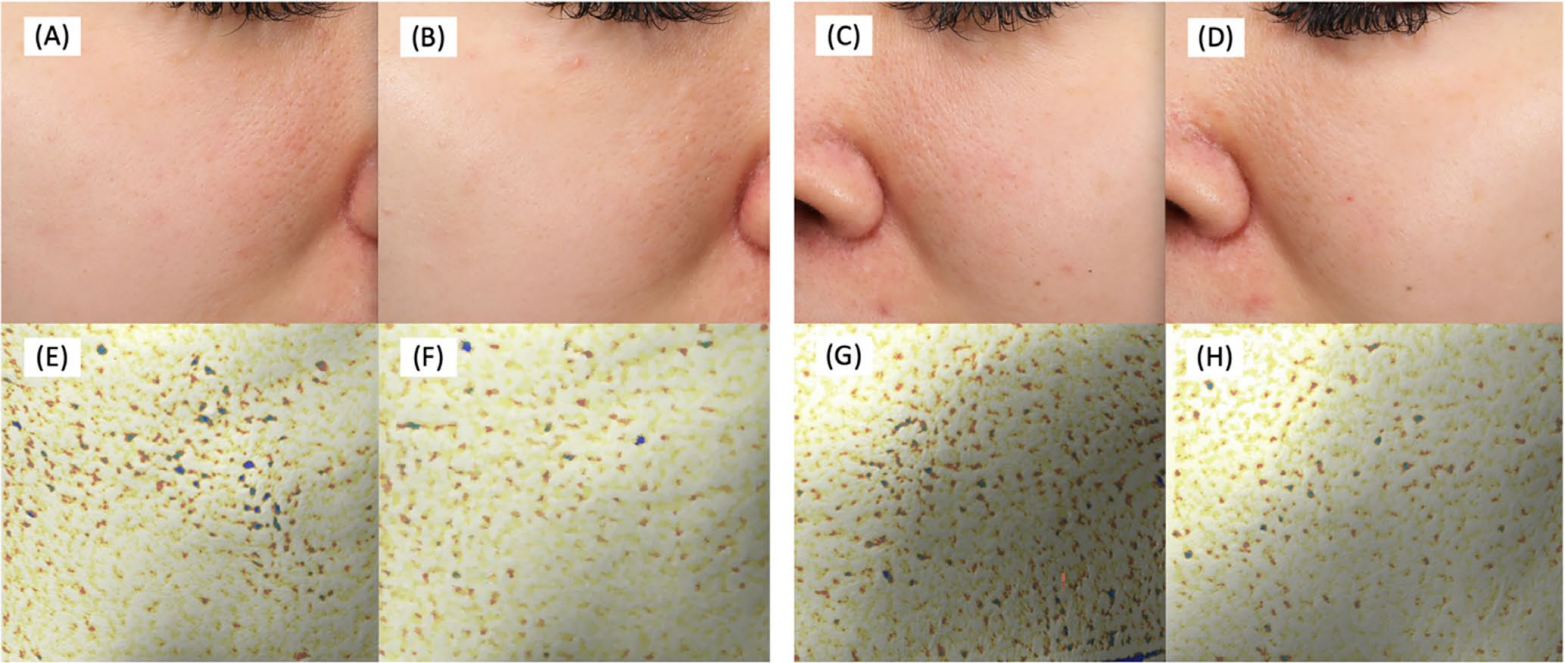
Clinical photographs of participants using VISIA and Antera 3D® at baseline and follow‐up visit at Week 32. (A, B) and (E, F) show the CPM‐HA20G treated side, while (C, D) and (G, H) show the CPM‐HA20 treated side.

By using Antera 3D, mean pore volume was calculated at baseline and every follow‐up visit. On average, pore volume decreased over time in both groups. The mean pore volume decreased by 0.02–0.58 mm^3^ on the CPM‐HA20G treated side. On the CPM‐HA20 treated side, the change in pore volume ranged from a reduction of 0.28 mm^3^ to an increase of 0.14 mm^3^ between Week 4 and Week 32. Over 32 weeks, the relative average pore volume reduction from baseline on the CPM‐HA20G treated side was 24.2% higher than on the CPM‐HA20 treated side (95% CI 1.4–47.1, *p* = 0.038). We observed a consistent trend of pore volume reduction during each follow‐up visit from the first injection session on the CPM‐HA20G treated side, and the mean pore volume reached its lowest point at Week 12. Mean pore volume is demonstrated in Figure 2. The photographs of the participants at baseline and Week 32 are shown in Figure 3.

### 3.3 Skin Hydration

**FIGURE 4:**
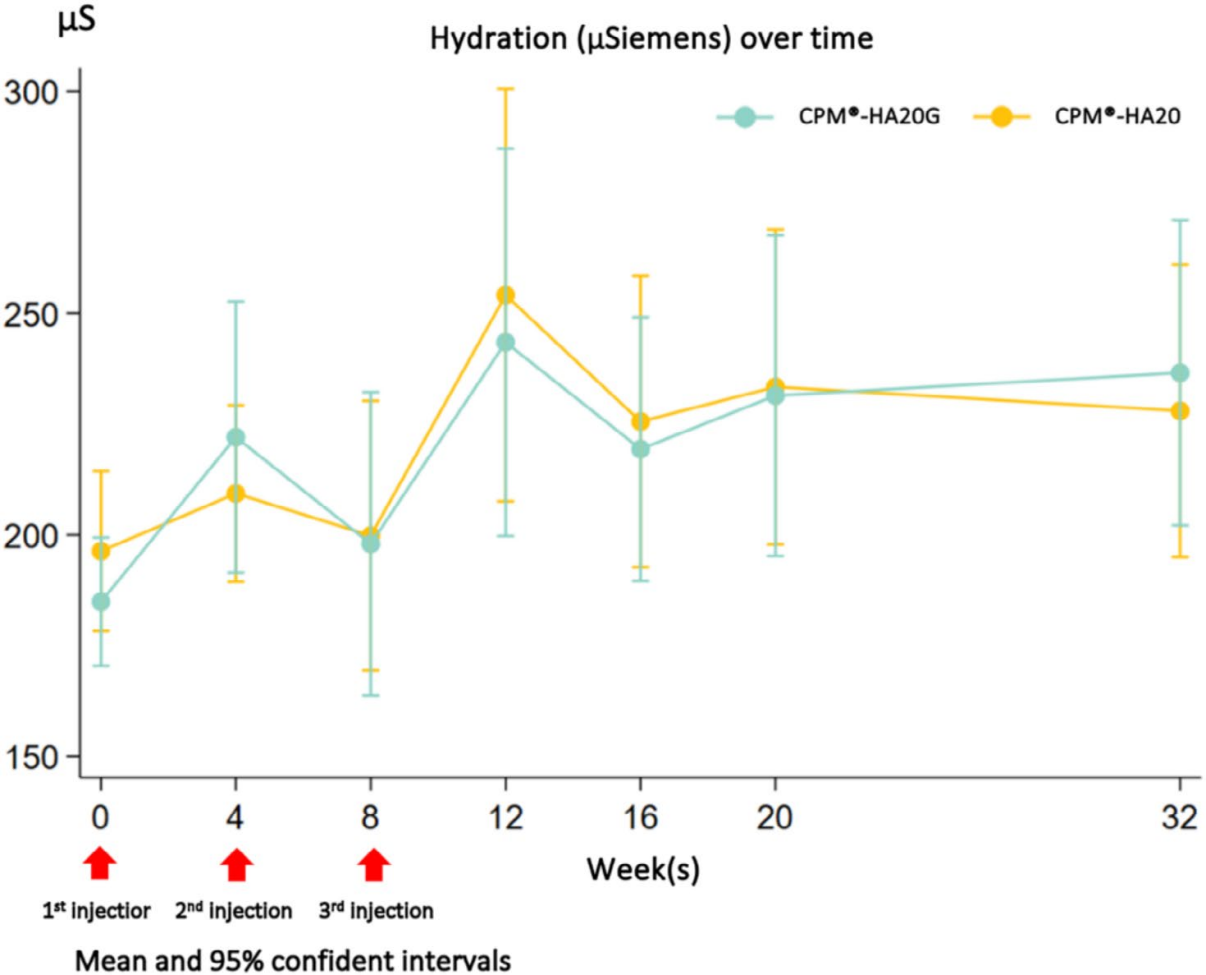
Mean hydration over time by Dermalab® combo.

Skin hydration increased throughout time in both groups. During Week 4 through Week 32, the average hydration increment was 15 to 62 μS in the CPM‐HA20G treated side and 4 to 38 μS in the CPM‐HA20 treated side. Over 32 weeks, the mean hydration increment from baseline on the CPM‐HA20G treated side was 6.4% higher than that on the CPM‐HA20 treated side (95% CI −0.8 to 13.6, *p* = 0.078). A significant difference in hydration increment was noted between both groups at Week 4, with the CPM‐HA20G treated side demonstrating an average hydration increment of 12.3% (95% CI 1.3–23.2, *p* = 0.029); there was also a marginally significant difference between the two groups at week 32, where the average hydration increment of the CPM‐HA20G treated side was 10.7% (95% CI −0.4 to 21.8, *p* = 0.06) higher than that of the CPM‐HA20 treated side, as shown in Figure 4.

### 3.4 Transepidermal Water Loss

There was no significant difference between the two groups on the change of TEWL from baseline and over 32 weeks (*p* = 0.32).

### 3.5 Skin Elasticity

Over time, there was no significant difference between CPM‐HA20G and CPM‐HA20 treated sides in terms of skin elasticity changed from baseline by measurement of viscoelasticity and retraction time (*p* = 0.80 and 0.39, respectively).

### 3.6 Subjective Assessment

#### 3.6.1 Pore Grading Score

For pore grading score evaluated by a one‐blinded dermatologist using the five‐point scale by criteria of visual assessment, the results showed no significant difference between two groups at baseline, Week 12 and Week 32 (Table 2). However, the CPM‐HA20G treated side had an average pore grading score reduction of two times higher than the CPM‐HA20 treated side when compared over time (95% CI 1.2–3.3, *p* = 0.009).

**TABLE 2:** Pore grading score evaluated by one‐blinded dermatologist using criteria of visual pore assessment.

Pore grading score	CPM‐HA20G, *n* (%)	CPM‐HA20, *n* (%)	*p*‐value (Fisher's exact test)
Baseline Week 0			0.68
0	0 (0)	0 (0)	
1	3 (10)	6 (21)	
2	18 (62)	18 (62)	
3	5 (17)	3 (10)	
4	3 (10)	2 (7)	
Follow‐up Week 12			0.91
0	1 (3.5)	0 (0)	
1	8 (28)	10 (34.5)	
2	14 (48)	13 (45)	
3	5 (17)	6 (21)	
4	1 (3.5)	0 (0)	
Follow‐up Week 32			1.00
0	1 (3.5)	2 (7)	
1	10 (34.5)	10 (36)	
2	14 (48)	13 (45)	
3	3 (10)	4 (14)	
4	1 (3.5)	0 (0)	

### 3.7 Satisfaction Assessment

Participant satisfaction evaluated by the seven‐point Likert GAIS showed an increase in the mean GAIS score from an average score of 1.2 and 1.1 at Week 4 to a score of 1.6–1.9 and 1.7–2.0 at Week 8 through Week 32 on CPM‐HA20G and CPM‐HA20 treated sides, respectively. There was no significant difference in patient satisfaction based on GAIS ratings between the CPM‐HA20G and CPM‐HA20 treated sides (OR 0.88, 95% CI 0.58–1.33, *p* = 0.54 and OR 0.94, 95% CI 0.61–2.15, *p* = 0.78, respectively).

### 3.8 Adverse Events

Overall, there was no significant difference between the two groups on the proportion of adverse events at each follow‐up visit. Most of the adverse events were papules and bruises immediately after the treatment sessions, but the severity of these adverse events was mild and tolerable, as shown in Table 3. One participant experienced delayed swelling around both medial cheeks, which subsided spontaneously after a week. The pain score was recorded after each treatment session, and there was no statistically significant difference in the mean pain score between both groups at each treatment session.

**TABLE 3:** Adverse events.

Follow‐up visits	Complications, *n* (%)		*p*‐value between groups (McNemar's exact test)
	CPM‐HA20, *n* (%)	CPM‐HA20G, *n* (%)	
Week 4			
Edema/swelling	17 (59)	17 (59)	1.000
Bruising/hematoma	19 (66)	20 (69)	1.000
Papules/nodules	0 (0)	1 (3)	1.000
Week 8			
Edema/swelling	25 (86)	25 (86)	1.000
Bruising/hematoma	17 (59)	14 (48)	1.000
Papules/nodules	1 (3)	1 (3)	1.000
Week 12			
Edema/swelling	22 (76)	21 (72)	1.000
Bruising/hematoma	15 (52)	14 (48)	1.000
Papules/nodules	4 (14)	3 (10)	1.000
Week 16			
Edema/swelling	0 (0)	0 (0)	1.000
Bruising/hematoma	0 (0)	0 (0)	1.000
Papules/nodules	0 (0)	0 (0)	1.000
Week 20			
Edema/swelling	1 (3)	1 (3)	1.000
Bruising/hematoma	0 (0)	0 (0)	1.000
Papules/nodules	0 (0)	0 (0)	1.000

## 4 Discussion

The aesthetic significance of enlarged facial pores is growing due to their association with skin surface evenness, one of the four emerging perceptual categories regarded as an indicator of skin quality [2]. Treatment is predicated on three primary pathogenesis factors: excessive sebum production, extracellular matrix degradation, and hair follicle size [6]. Multiple studies on HA‐based dermal fillers have demonstrated their efficacy in reducing enlarged facial pores, either as a monotherapy or in conjunction with other procedures. In our study, it was shown that both the CPM‐HA20G and CPM‐HA20 treated sides were effective at reducing pore volume throughout the 8‐month study period, with a maximum efficacy at Week 12. This result demonstrated that HA filler was effective at diminishing enlarged facial pores, which was consistent with the findings of Qian et al. It was demonstrated that an intradermal injection of low molecular weight HA substantially reduced facial pore size and enhanced skin texture, with a 40.03% improvement rate and a 92.8% overall satisfaction rate after 6 months of injection [11]. These results could be explained by the fact that collagen fragmentation from aging or photodamaged skin causes fibroblast collapse, decreased collagen synthesis, and elastic fiber degradation, all of which lead to increased pore size. Injecting HA filler replenishes the extracellular dermal scaffold while improving enlarged facial pores. Furthermore, micropuncture injections of soft‐tissue fillers enhance skin elasticity and reduce surface roughness [17, 18]. However, our study showed that the CPM‐HA20G treated side showed a significantly higher efficacy in this regard than the CPM‐HA20 treated side. There were two possible explanations for this outcome. First, both products contained HA. It is widely recognized that HA‐based dermal fillers not only restore the extracellular matrix space caused by collagen fragmentation, but also indirectly stimulate the production of collagen and elastic fibers by inducing mechanical stretching of fibroblasts [18, 19, 20, 21]. Second, CPM‐HA20G contains glycerol, which functions as a potent humectant. Multiple studies have substantiated the efficacy of CPM‐HA20G in improving skin hydration and the viscoelastic properties of the skin, including skin elasticity, firmness, and fatigue. All these elements contribute to improving the structural support of follicular structures [13, 14], and our study indicates the incremental benefit of glycerol in CPM‐HA20G for enlarged facial pores as compared to formulations that contain only HA.

In terms of skin hydration, both the CPM‐HA20G and CPM‐HA20 treated sides demonstrated an overall improvement over time. Following the third treatment session, the skin hydration increased to its highest point at Week 12. A consistent trend in skin hydration was observed from baseline at Week 0 throughout Week 32. It was consistent with the findings of the study by Hertz‐Kleptow et al. which demonstrated an improvement in skin hydration from baseline up to 36 weeks following the three sessions of CPM‐HA20G in the lower cheek at 4 weeks apart [13]. For other biophysical parameters, this study found that there was no significant difference in skin elasticity demonstrated by viscoelasticity property and skin retraction time between both groups. This finding could be explained by the same HA concentration in both treatment groups, while glycerol may exert an insignificant effect on the alteration of skin elasticity.

For subjective evaluation, our study demonstrated a statistically significant decrease in the overall pore grading score, graded by the blinded dermatologist, from baseline to Week 32 in the CPM‐HA20G treated side compared to the CPM‐HA20 treated side. The finding complied with the mean pore volume reduction change described in the aforementioned data. Participant‐reported satisfaction, using the seven‐point Likert GAIS, showed no significant difference between the two groups in every follow‐up visit.

Overall, treatment‐related adverse events were generally mild and tolerable. The adverse events included injection site pain, swelling, bruising, or hematoma. All these adverse events resolved spontaneously within 1–2 weeks without any long‐term sequelae.

There are some limitations in our study. First, the small sample size could have resulted in an underestimation of significant differences in skin hydration improvement, TEWL, viscoelasticity measurement, and subjective assessment by both physician and participants. Second, we did not assess our participants' sebum secretion status, which is one of the main factors impacting facial pore size. Assessing the level of sebum secretion could potentially provide further elucidation about the mechanism underlying enlarged facial pores in both treatment groups. Finally, unlike earlier studies, ours did not classify participants based on their degree of skin dryness. This led to a lack of information on outcomes in each specific group.

In conclusion, 3‐monthly injections of both CPM‐HA20G and CPM‐HA20 were effective in reducing enlarged face pores and improving skin hydration status. However, CPM‐HA20G demonstrated superior efficacy in terms of minimizing enlarged facial pores. Furthermore, patient satisfaction was comparable, with acceptable downtime in both treatment groups. Prospective studies regarding different injection techniques and protocols should be further investigated for the optimum result.

## Author Contributions

**Thanachat Rutnumnoi:** conducting patient evaluation, collecting data, writing, and revising the manuscript. **Pasita Palakornkitti:** conducting patient evaluation and collecting data. **Tanaporn Anuntrangsee:** conducting patient evaluation and collecting data. **Tanat Yongpisarn:** conducting patient evaluation and collecting data. **Nawara Sakpuwadol:** conducting patient evaluation and collecting data. **Vasanop Vachiramon:** conducting patient evaluation, writing, reviewing, and revising the manuscript.

## Ethics Statement

This study protocol conformed to the guidelines of the Declaration of Helsinki and was approved by the Institutional Review Board of Human Rights Related to Research involving Human Subjects, Faculty of Medicine Ramathibodi Hospital, Mahidol University, Thailand.

## Conflicts of Interest

The authors declare no conflicts of interest.

## Data Availability

The data that support the findings of this study are available from the corresponding author upon reasonable request.
